# Serum 25-hydroxyvitamin D levels and mortality risk in patients with liver cirrhosis: a protocol for a systematic review and meta-analysis of observational studies

**DOI:** 10.1186/s13643-019-0988-6

**Published:** 2019-03-23

**Authors:** Désirée Völker, Frank Grünhage, Stefan Wagenpfeil, Frank Lammert, Caroline S. Stokes

**Affiliations:** 1 Department of Medicine II, Saarland University Medical Center, Saarland University, Kirrberger Str. 100, 66421 Homburg, Germany; 2 Department of Internal Medicine, Grevenbroich St. Elisabeth Hospital, Grevenbroich, Germany; 3 Institute of Medical Biometry, Epidemiology and Medical Informatics, Saarland University, Campus Homburg, Homburg, Germany

**Keywords:** Calcidiol, Death, Deficiency, Liver disease, Survival

## Abstract

**Background:**

Liver cirrhosis represents a substantial global burden in terms of morbidity and mortality. Observational studies have reported an increased risk of death with low circulating 25-hydroxyvitamin D concentrations in such patients. Because the occurrence of inadequate vitamin D status is very common in patients with liver cirrhosis, the aim of this study is to conduct a meta-analysis of observational studies in such patients to assess whether vitamin D deficiency increases their risk of mortality.

**Methods:**

We will search electronic databases (MEDLINE, Embase, Web of Science, CENTRAL and Google Scholar from time of inception until now), conference proceedings and conduct manual searches to identify studies eligible for inclusion. There will be no restrictions based on publication status or language, and the meta-analysis will be reported in accordance with the MOOSE guidelines. We will employ random-effects meta-analysis to assess the relationship between vitamin D deficiency and risk of mortality. Quality of studies will be judged using the Newcastle-Ottawa scale, and between-trial heterogeneity will be evaluated by means of subgroup and sensitivity analyses.

**Discussion:**

The study will assess the effects of serum 25-hydroxyvitamin D concentrations on mortality in patients with liver cirrhosis. The results will be published in a high-quality peer-reviewed journal.

**Systematic review registration:**

Prospero CRD42016052007.

**Electronic supplementary material:**

The online version of this article (10.1186/s13643-019-0988-6) contains supplementary material, which is available to authorized users.

## Background

### Description of the condition

Liver cirrhosis represents a substantial global burden in terms of morbidity and mortality, with over one million deaths documented in 2010, as compared to 676,000 in 1980 [1]. While the highest rates are found in south-eastern and north-eastern Europe, the overall mortality associated with liver cirrhosis illustrates a significant increase in many European countries. The most common causes of liver cirrhosis include extensive alcohol consumption, viral hepatitis and metabolic syndrome, all of which are, to a great extent, modifiable in terms of prevention and treatment [2–4].

Liver cirrhosis is defined as the destruction of the (micro)architecture of the liver by excessive deposits of extracellular matrix components eventually resulting in loss of liver function [5]. The diagnostic procedures include imaging with ultrasonography, CT or MRI, surrogate markers of liver inflammation such as aminotransferases and histological examination by liver biopsy [5]. There are two different classification systems for the characterisation and assessment of clinical severity of liver disease that provides prognostic information for patients with cirrhosis. Both the Child-Pugh score and the MELD (Model for End-Stage Liver Disease) can be used to predict survival of patients with cirrhosis [6].

### What is the role of vitamin D in liver cirrhosis?

An association of vitamin D deficiency in patients with liver cirrhosis and disease prognosis has consistently been documented [7–10]. The prevalence of insufficient vitamin D concentrations (< 30 ng/ml) occurs in the majority of patients with chronic liver disease (CLD), and vitamin D deficiency (< 20 ng/ml) in approximately two thirds of patients [9, 10]. A study of 118 patients showed that severe vitamin D deficiency, defined as vitamin D levels < 7 ng/ml, was more common among patients with cirrhosis, as compared to those without (29.5% vs. 14.1%) [10]. Vitamin D deficiency is defined by serum 25-hydroxyvitamin D (25(OH)D) levels < 20 ng/ml (50 nmol/l) [11, 12]; however, optimum levels are still debated. While the Institute of Medicine (IOM) of the National Academies in the USA states that serum 25(OH)D concentrations of 20 ng/ml are adequate, the Endocrine Society suggests that levels of 30 ng/ml (75 nmol/l) are preferable [13, 14].

Several studies [15, 16] have investigated the association of vitamin D and increased risk of all-cause mortality, with some depicting a reverse J-shaped association between serum concentrations of 25(OH)D and mortality [16]. A meta-analysis based on the general population [17] revealed a nonlinear decline in overall mortality as 25(OH)D concentrations increased. This association was subsequently explored by a meta-analysis [15] of individual participant data from 26,018 men and women in Europe and the USA that found a consistent association between low serum 25(OH)D levels and mortality.

Furthermore, a large meta-analysis of observational cohorts and randomised intervention studies [18] with 849,412 and 30,716 participants, respectively, reported an inverse correlation between serum 25(OH)D levels and risk of death due to cardiovascular disease, cancer and other causes [18]. According to their investigation, each 10 ng/ml decline in 25-hydroxyvitamin D concentration was associated with a 16% increased risk of all-cause mortality. In contrast, Allan et al. [19] analysed whether the most frequently studied associations between vitamin D deficiency and outcomes such as falls prevention, cancer treatment options and overall mortality could be verified. They argue that for most of the studies, mortality was a secondary outcome instead of the main endpoint and that relative mortality reduction actually ranged around 5%.

Research studies investigating the effect of vitamin D supplementation in patients with CLD are controversial [7, 20, 21]. For instance, a recent case-control study [21] including 101 vitamin D deficient patients with decompensated cirrhosis showed that treatment with cholecalciferol and calcium for 6 months resulted in greater survival in the treatment group (69% vs. 64%); however, their results were not significant. In contrast, a recent review by Bjelakovic et al. [22] analysing RCTs with 1034 participants could not find any evidence for the effect of vitamin D supplementation on all-cause mortality or liver-related mortality for patients with CLD.

Finally, the VITAL study—a population-based RCT [23]—including 25,871 participants reported that administration of vitamin D3 (2000 IU/day) for an average of 5.3 years did not reduce risk of cancer or major cardiovascular events. Such conflicting findings may be partly influenced by baseline serum 25(OH)D concentrations [24], which were at approximately 30 ng/ml in the VITAL study. Moreover, different 25(OH)D quantification methods introduce variability in estimated concentrations [25]. The specific patient population might also have an influential role.

There are several complications associated with liver cirrhosis, such as spontaneous bacterial peritonitis or an increased risk of infections, in which vitamin D may play a role. Anty et al. [26] discovered an association between vitamin D deficiency and the occurrence of bacterial infections in patients with liver cirrhosis. Polymorphisms of genes implicated in vitamin D metabolism and transport have been shown to influence serum 25(OH)D concentrations in healthy populations. In liver patients, we previously observed an association between serum 25(OH)D concentrations and *CYP2R1* and *DHCR7* polymorphisms, as well as with fibrosis severity [27]. Therefore, the presence of such polymorphisms could affect the patients’ vitamin D baseline levels, in addition to their response to treatment.

### Aim and hypothesis

Given the pertinent findings from the recent meta-analyses on vitamin D and mortality, the goal of the meta-analysis herein is to determine whether vitamin D deficiency is associated with increased mortality in patients with liver cirrhosis. Specifically, we aim to compare adults with liver cirrhosis and low serum 25(OH)D concentrations to adults with cirrhosis and higher serum 25(OH)D levels, to determine whether the former is associated with increased mortality in observational studies with a minimum follow-up period of 3 months.

## Methods

### Research objectives

The research objective is to conduct a systematic review and meta-analysis of observational studies to investigate the association between vitamin D concentrations and risk of mortality in patients with liver cirrhosis. The meta-analysis has been registered on Prospero (CRD42016052007) and will be reported in accordance with the MOOSE guidelines [28]. This protocol is based on the PRISMA-P guidelines [29] (see Additional file 1).

### Type of studies

Observational prospective and retrospective cohort studies, irrespective of publication status or language will be included in the primary analysis. To be eligible for inclusion, studies must have investigated the association between serum vitamin D concentrations and mortality in patients with liver cirrhosis.

### Type of participants

We will include studies of adult patients with liver cirrhosis, of all stages (regardless of the underlying liver disease aetiology, age, sex and ethnicity group). Diagnosis of liver cirrhosis will be based on either histology, imaging or unequivocal signs of cirrhosis such as oesophageal varices, hepatic encephalopathy or ascites, as defined by the authors.

### Main outcome

Our primary outcome is all-cause mortality (defined as deaths from any cause) at maximum duration of follow-up.

### Search strategy

MEDLINE, Embase, Web of Science, CENTRAL (Cochrane Central Register of Controlled Trials) and Google Scholar will be searched from the time of inception until the present. A structured search strategy (an example is presented in the Additional file 2) will include clearly defined search terms (including MESH terms) for searching relevant articles (such as cholecalciferol, vitamin D, 25(OH)D, calcidiol) and outcomes (death, liver cirrhosis, mortality) without any language or publication restrictions. Additionally, reference lists of included articles, as well as conference proceedings from the following organisations: AASLD (American Association for the Study of Liver Diseases) and EASL (European Association for the Study of the Liver), will be scanned for further references. For conference proceedings, two authors (DV and CSS) will independently scan all titles and abstracts from 2010 until the present with the use of the abovementioned search terms. We will also contact principle authors of studies when we come across relevant abstracts. Studies published in any language other than English will be translated by the authors (German, Greek) or professionally, before inclusion. The search will be conducted by two authors (DV and CSS) individually, and the results will be compared and screened as stated below. One author (CSS) has previous experience with published meta-analyses and the other (DV) received training by a librarian on how to conduct these searches.

### Data collection and analysis

#### Selection of studies

All potentially relevant titles and abstracts will be screened by two authors (DV and CSS) and evaluated according to the predefined eligibility criteria stated below. For studies that meet the inclusion criteria, full-text articles will be retrieved and data extraction will be performed. Full-text articles will also be retrieved for studies with insufficient information in the abstract, for the evaluation of inclusion and exclusion criteria. Any disagreement between the authors will be resolved by discussion involving a third neutral party (FL) or a fourth (FG) until a consensus is reached. If studies are described in more than one publication, we will select the reference with the longest duration of follow-up as the primary reference.

#### Eligibility criteria

The analysis will focus on adults (> 18 years) with liver cirrhosis, in whom serum 25(OH)D concentrations are measured. Patients with low vs. high serum 25(OH)D concentrations will be compared when followed up for mortality for a minimum of 3 months, based on data from observational studies. Studies that report the effect of vitamin D concentrations on hepatic diseases but not on mortality will be excluded.

### Data extraction

Data will be extracted by two authors (DV and CSS) using a pre-formatted data collection form, and any disagreements will be resolved through discussion and will include a third author (FG). The following data will be collected: general information (e.g. sample size, study design, length of follow-up) and patient population (age at baseline, sex, ethnicity, underlying hepatic disease and classification according to Child-Pugh or MELD score, decompensated vs. compensated liver cirrhosis, medication or current therapy used and sociodemographic and lifestyle determinants such as physical activity, body mass index, smoking and social status), vitamin D assay method, outcomes such as mortality rates of participants during follow-up, number and type of complications associated with liver cirrhosis and if possible person-time data, incidence rates and hazard ratios. We will also convert all serum 25(OH)D levels to nanogrammes per millilitre (ng/ml) by dividing by 2496 when reported in nanomoles per litre (nmol/l) [17]. We will also collect data for assessment of bias, as outlined below, and summarise the characteristics of included studies in a tabular format. We will contact authors during the data extraction for clarification or further information, as needed.

### Assessment of risk of bias

Given that the studies are of observational nature, they will be evaluated with the Newcastle-Ottawa scale [30] (see Additional file 3). This scale system evaluates a study in three domains: selection of participants, comparability of study groups and the identification of outcomes of interest. A star system is used with a maximum of nine stars. Correspondingly, studies are classed as having a low, moderate high risk of bias, depending on the number of stars awarded.

### Data synthesis

We will perform the meta-analysis with StatsDirect 3.0 software using different statistical tools. For dichotomous data associated with vitamin D concentrations, relative risks with 95% confidence intervals (CI) will be calculated and presented. Furthermore, we also include the incidence rate ratio and hazard ratios as summary measures when available. Continuous data will be presented using mean differences and 95% CI. For pooling data, the random effects model is used [31]. Possible heterogeneity between study populations is assessed using Cochran’s Q test and I-squared measure. Furthermore, we present funnel plots and explorative, two-sided *p* values resulting from Egger’s test for funnel plot asymmetry to account for possible publication bias. We will assess the overall strength of the body of evidence using the Grading of Recommendation Assessment, Development and Evaluation (GRADE) tool.

Furthermore, U-shaped or inverse J-shaped associations between circulating 25(OH)D concentrations and total mortality have been reported in some observational studies, therefore non-linear associations between 25(OH)D and mortality in patients with liver cirrhosis will be assessed, if possible. For example, if the necessary information is available in the studies that will be included in the meta-analysis, mortality will be evaluated according to trichotomised vitamin D concentrations (based on low, medium and high serum levels).

### Dealing with missing data

For missing data due to dropouts or for patients lost to follow-up, reasons for dropout will be collected, and if no information is given, authors will be contacted for more information. Additionally, authors will be contacted if data on outcomes are missing. We will conduct a systematic analysis of the missing data, with for example the inclusion of a worst-case scenario analysis, assuming all dropouts and patients lost to follow-up died [32].

### Assessment of heterogeneity

The *I*^2^ statistic will be included, which is a formal test of statistical heterogeneity and indicates the expected between-study heterogeneity. Specifically, the *I*^2^ statistic will be used to express the percentage of the total variation across studies resulting from heterogeneity [33]. These will be grouped using the following values: 0% to 40% might not be important, 30% to 60% may represent moderate heterogeneity, 50% to 90% may represent substantial heterogeneity, and 75% to 100% considerable heterogeneity [34]. To investigate possible sources of heterogeneity, we will carry out subgroup analyses.

### Subgroup analysis

Studies with similar features will be grouped together allowing for further comparisons. For example, we will compare outcomes for patients with different vitamin D status, e.g. serum 25(OH)D concentrations < and > 30 and < and > 20 ng/ml, in addition to < and > 10 ng/ml.

We will further compare all-cause mortality in compensated cirrhosis (which is broadly defined as the absence of complications, including no varices [35, 36]) vs. decompensated cirrhosis (defined using clinical signs: ascites, bleeding, hepatic encephalopathy, jaundice) [37] and based on the stage of cirrhosis, as defined by the Child-Pugh score [38] or where possible, by the assessment of complications such as hepatic encephalopathy (defined as brain dysfunction caused by liver insufficiency and/or portosystemic shunting [39]), variceal bleeding due to portal hypertension [35], ascites (accumulation of intraperitoneal liquid examined by sonography and computed tomography [40, 41]), or the presence of infections [42]. Where possible, we will also compare the length of follow-up and cirrhosis aetiology. In order to further analyse heterogeneity, meta-regression analysis will be performed, and different explanatory variables, such as the Charlson comorbidity index (CCI) [43] and other prediction parameters suggested by Haj et al. [38] including hepatic venous pressure gradient (HVPG) measurements, Child-Pugh score or MELD score, will be used.

In the presence of a sufficient number of studies, we will also compare the assay methods for vitamin D measurement, which have previously shown to lead to variability in findings in meta-analyses assessing the relationships between vitamin D and mortality in the general population [17]. If it is not possible to quantitatively synthesise the data in order to conduct subgroup analyses, we will provide a descriptive summary for data related to the stages of cirrhosis and complications for the included studies.

### Sensitivity analysis

In order to assess the robustness of our findings, sensitivity analysis will be carried out to determine the influence of different factors on effect size. Such factors will include publication bias which can be assessed using funnel plots with the Egger test for symmetry [44]. Other factors include the duration of follow-up and studies judged as having a high or low risk of bias. We will also carry out a worst-case scenario based on dropouts and patients lost to follow-up (as described above).

## Discussion

The study will evaluate the effects of serum 25(OH)D concentrations on survival in patients with liver cirrhosis. This provides the opportunity to improve the knowledge on risk factors for this patient group and may also highlight the need for vitamin D supplementation studies in this area. The results will be presented at international and national conferences and published in a high-quality peer-reviewed journal.

## Additional files

Additional file 1: PRISMA-P (Preferred Reporting Items for Systematic review and Meta-Analysis Protocols) checklist. (DOCX 18 kb)

Additional file 2: Search strategy. (DOCX 15 kb)

Additional file 3: Newcastle-Ottawa quality assessment scale for cohort studies (NOS). (DOCX 15 kb)

## Abbreviations

AASLD: American Association for the Study of Liver Diseases
CCI: Charlson comorbidity index
CI: Confidence interval
CLD: Chronic liver disease
CT: Computer tomography
CYP2R1: Cytochrome P450, family 2, subfamily R1
DBP: Vitamin D binding protein
DHCR7: Encoding 7-dehydrocholesterol reductase
EASL: European Association for the Study of the Liver
GRADE: Grading of Recommendation Assessment, Development and Evaluation
HVPG: Hepatic venous pressure gradient
MELD: Model for End-Stage Liver Disease
MRI: Magnetic resonance imaging
VDR: Vitamin D receptor
