# Supplementary material for: Serum 25-hydroxyvitamin D levels and mortality risk in patients with liver cirrhosis: a protocol for a systematic review and meta-analysis of observational studies
Source: Syst Rev. 2019 Mar 23;8:73. doi: 10.1186/s13643-019-0988-6 (PMC6431049; doi:10.1186/s13643-019-0988-6)
Supplement: Supplementary file 1 — PRISMA-P (Preferred Reporting Items for Systematic review and Meta-Analysis Protocols) checklist. (DOCX 18 kb) [file 13643_2019_988_MOESM1_ESM.docx]

**Additional file 1: PRISMA-P (Preferred Reporting Items for Systematic review and Meta-Analysis Protocols) checklist**

**Serum 25-hydroxyvitamin D levels and mortality risk in patients with liver cirrhosis: a protocol for a systematic review and meta-analysis of observational studies**

**ADMINISTRATIVE INFORMATION**

Title

Identification: Protocol of a systematic review

Registration: Prospero CRD42016052007

Authors

Contact: Désirée Völker (DV), Frank Grünhage (FG), Stefan Wagenpfeil (SW), Frank Lammert (FL), Caroline S. Stokes (CS)

Contributions: DV and CSS participated in the conception and design of this protocol. SW provided statistical advice for the design. DV and CSS prepared the draft and all authors reviewed the manuscript and approved the final version.

**INTRODUCTION**

Rationale

Liver cirrhosis represents a substantial global burden in terms of morbidity and mortality. Observational studies have reported an increased risk of death with low circulating 25-hydroxyvitamin D concentrations in such patients. Because the occurrence of inadequate vitamin D status is very common in patients with liver cirrhosis, the aim of this study is to conduct a meta-analysis of observational studies in such patients to assess whether vitamin D deficiency increases their risk of death of mortality.

Objectives

The aim of the meta-analysis is to determine whether vitamin D deficiency is associated with increased mortality in patients with liver cirrhosis.

**METHODS**

Eligibility criteria

Observational prospective and retrospective cohort studies, no language restriction, from time of inception until today.

Information sources

MEDLINE, EMBASE, Google Scholar, Web of Science, CENTRAL (Cochrane Central Register of Controlled Trials), conference proceedings of AASLD and EASL.

Search strategy

see Supplementary Material

Study records

Data management/Selection process: All potentially relevant titles and abstracts will be screened by two authors (DV and CSS) and evaluated according to predefined eligibility criteria. Any disagreement between the authors will be resolved by discussion involving a third neutral party (FL) or a fourth (FG) until a consensus is reached.

Data collection process: Data will be extracted by two authors (DV and CSS) using a pre-formatted data collection form, and any disagreements will be resolved through discussion and will include a third author (FL) or a fourth if needed (FG).

Data items: general information (sample size, study design), patient population (age at baseline, sex, race, underlying hepatic disease), vitamin D assay method, outcomes (mortality rates of participants), number and type of complications, person-time data, incidence rates, hazard ratios.

Outcomes and prioritization

Primary: All-cause mortality (defined as deaths from any cause) at maximum duration of follow-up.

Risk of bias in individual studies

Studies will be evaluated with the Newcastle-Ottawa Scale.

Data synthesis

Relative risks with 95% CI will be calculated for dichotomous data. We also include the incidence rate ratio and hazard ratios as summary measures. Continuous data will be presented using mean differences and 95% CI. For pooling data the random effects model is used. Possible heterogeneity between study populations is assessed using Cochran's Q-Test and I-squared measure. Furthermore, we present funnel plots and explorative, two-sided p-values resulting from Egger's test for funnel plot asymmetry to account for possible publication bias.

Confidence in cumulative evidence

We will assess the overall strength of the body of evidence using the Grading of Recommendation Assessment, Development and Evaluation (GRADE) tool.

Reference: Shamseer L, Moher D, Clarke M, et al. Preferred reporting items for systematic review and meta-analysis protocols (prisma-p) 2015: Elaboration and explanation. BMJ. 2015. doi:10.1136/bmj.g7647
