# Supplementary material for: Serum 25-hydroxyvitamin D levels and mortality risk in patients with liver cirrhosis: a protocol for a systematic review and meta-analysis of observational studies
Source: Syst Rev. 2019 Mar 23;8:73. doi: 10.1186/s13643-019-0988-6 (PMC6431049; doi:10.1186/s13643-019-0988-6)
Supplement: Supplementary file 2 — Search strategy. (DOCX 15 kb) [file 13643_2019_988_MOESM2_ESM.docx]

Additional file 2: Search strategy

We will identify relevant observational prospective and retrospective studies through electronic searches using MEDLINE, Web of Science, EMBASE, CENTRAL (Cochrane Central Register of Controlled Trials) and Google Scholar published from time of inception until the present and without any language or publication restriction. Furthermore we will search conference proceedings of the Amercian Association for the Study of Liver Diseases (AASLD) and European Association for the Study of the Liver (EASL) from 2010 until the present. Additionally, we will scan reference lists of articles identified for all relevant studies.

(i) MEDLINE strategy for identifying relevant exposures:

(vitamin D OR vitamin d OR 25-hydroxyvitamin D OR 25(OH)D OR cholecalciferol OR colecalciferol OR calcidiol)

(deficiency OR low levels)

(ii) MEDLINE strategy for identifying relevant outcomes:

(mortality OR death OR died OR expired OR prognosis)

(iii) MEDLINE strategy for identifying relevant population:

(liver disease OR cirrhosis OR chronic liver disease OR alcoholic liver disease)

All parts (i – iii) will be combined using ‘AND’ to search the database.
