# Supplementary material for: Serum 25-hydroxyvitamin D levels and mortality risk in patients with liver cirrhosis: a protocol for a systematic review and meta-analysis of observational studies
Source: Syst Rev. 2019 Mar 23;8:73. doi: 10.1186/s13643-019-0988-6 (PMC6431049; doi:10.1186/s13643-019-0988-6)
Supplement: Supplementary file 3 — Newcastle-Ottawa quality assessment scale for cohort studies (NOS). (DOCX 15 kb) [file 13643_2019_988_MOESM3_ESM.docx]

**Additional file 3: Newcastle - Ottawa Quality Assessment Scale for Cohort Studies (NOS)**

**Selection**

Representativeness of the exposed cohort

1. Truly representative of the average patient with liver cirrhosis in the community
2. Somewhat representative of the average patient with liver cirrhosis in the community
3. Selected group of users
4. No description of the derivation of the cohort

Selection of the non-exposed cohort

1. Drawn from the same community as the exposed cohort
2. Drawn from a different source
3. No description of the derivation of the non-exposed cohort

Ascertainment of exposure

1. Secure record: serum 25-hydroxyvitamin D levels via patient record and/or blood test result
2. Structured interview
3. Written self report
4. No description

Demonstration that outcome of interest was not present at start of study

1. Yes (patient alive)
2. No

**Comparability**

Comparability of cohorts on the basis of the design or analysis

1. Study controls for confounders, losses to follow-up and patients receiving liver transplants
2. Study controls for any additional factor, such as complications associated with decompensated cirrhosis such as HE, bleeding, ascites

**Outcome**

Assessment of outcome

1. Independent blind assessment
2. Record linkage
3. Self report
4. No description

Was follow-up long enough for outcomes to occur

1. Yes (follow-up > 3 months)
2. No

Adequacy of follow-up of cohorts

1. Complete follow-up – all subjects accounted for (death as event)
2. Subjects lost to follow-up unlikely to introduce bias – small number lost < 20%
3. Follow-up rate < 80% and no description of those lost
4. No description or unclear
